# Editorial: Biomarkers and Pathogenesis of Alpha-Synuclein in Parkinson's Disease

**DOI:** 10.3389/fnagi.2021.776873

**Published:** 2021-10-05

**Authors:** Puyu Li, Pingyi Xu, Jun Liu

**Affiliations:** Department of Neurology, Ruijin Hospital Afflicted to Shanghai Jiaotong University School of Medicine, Shanghai, China

**Keywords:** alpha-synuclein (α-synuclein), Parkinson's disease, biomarker (BM), pathogenesis (nervous system), predictor

The recognition of Alpha-Synuclein (α-Syn) as an essential player in the pathobiology of human neurodegenerative diseases has spurred substantial efforts to understand its biochemical and pathological function. This *Frontiers in Aging Neuroscience* Topic contains a series of high-quality original articles and updated reviews covering recent 2-year progression in various aspects of α-Syn expressions in blood, saliva, and cerebrospinal fluid (CSF), and pathological processes in regulating human neurodegenerative disorders as well as its feasibility as an early predictive biomarker. Parkinson's disease (PD) is a typical disease in α-synucleinopathies, presenting dominantly with clinical symptoms of motor impairment. PD is characterized by progressive chronic neurodegeneration and loss of dopaminergic (DA) neurons in brain, mainly substantia nigra. Meanwhile, dementia with Lewy bodies (DLB), and multiple system atrophy (MSA) belong to this devastating neurodegenerative category. Currently, PD cannot be completely cured or reversed, and existing treatment strategies have limited efficacy in the early stages. Therefore, there is an imperative need to clarify the pathogenesis of PD and find early identifications of it. However, the detailed etiology and pathogenesis remain no consensus. This Research Topic aims to generate a multidimensional discussion linking clinical analysis with basic experiment unveiling the fundamental mechanisms of PD. Then filtrate the current effective biomarkers for preclinical detection and clinical evaluation on this basis. As a result, building a predictive model contributes to a deeper understanding of the mechanism of PD, so as to better guide clinical applications.

Various attempts made by the researchers to develop preclinical and clinical investigations encompassing genetics, immunology, microbiology, neuroimaging, and neurophysiology to clarify the molecular mechanisms and identify useful biomarkers for the disease diagnosis. There are two valuable systematic reviews on this topic, which comprehensively describe the current research status. In the first place, Du et al. summarizes useful biomarkers for the diagnosis of PD, as well as how the biomarkers α-Syn could potentially be used as a biomarker for PD step by step from genetics, immunology, fluid and tissue, imaging, as well as neurophysiology. As a general overview, Ganguly et al. also evaluate the overall effectiveness of α-Syn in forecasting PD progression. In view of the overwhelming importance of α-Syn in the pathogenesis, a detailed analysis is then made of various studies to establish the biomarker potential of this protein in PD. Knowledge of the α-Syn-related novel biomarkers for preclinical detection and clinical evaluation serve to identify the disease-predictive biomarkers more effectively, which are critical for precise diagnosis and therapeutic approaches.

Early warning biomarkers have become a hot topic recently due to the need for diagnosis and treatment in forepart. Inchoate detection and differential diagnosis of parkinsonism-type MSA (MSA-P) and PD are in great clinical need, as aging can lead to many diseases that are difficult to detect at early stage. This urgent issue is also addressed by Li et al. in this case by studying the role of neuroimaging in the diagnosis of MSA-P. Using a novel magnetic resonance technology, amide proton transfer (APT) imaging, which is a well-studied chemical exchange saturation transfer imaging for its sensitivity to mobile protons and peptides in tissues. Next, Xian et al. demonstrate the diagnostic value of co-registration analysis of ^18^F-flurodeoxyglucose (^18^F-FDG) and ^18^F-Dopa positron emission tomography (PET) for differentiating MSA-P from PD. Segmentation analysis of the rabbit-shaped appearance of the striatum segment of PET images not only could distinguish PD and MSA-P patients from healthy controls (HCs).

Rapid eye movement sleep behavior disorder (RBD) is marked by repeated nocturnal sleep-related normal skeletal muscle atonia with remarkable motor disturbances and dream enactment behaviors, and the existence of RBD can be considered as an omnipotent precursor to PD. Therefore, scholars have started with exploring the interconnected mechanisms between RBD and PD and the course progression to identify early predictive indicators of PD. The article by Si et al. assess the clinical significance of magnetic resonance imaging (MRI)-visible enlarged perivascular space (EPVS) in patients with idiopathic RBD (iRBD) and PD. Their study indicates that iRBD patients had significantly greater EPVS burdens than PD patients. Specifically, higher EPVS burdens in centrum semiovale (CSO) and basal ganglia might be independent risk factors for iRBD, and the higher the value the more severe the clinical symptoms. Then, Chen et al. combine clinical assessments, dopamine transporter (DAT) imaging data, CSF and genetic data of PD patients to minister to develop a prognostic model for predicting mild cognitive impairment (MCI) in PD-RBD patients. As CI is one of the most common non-motor symptoms in PD, incidence of CI in PD patients increases as the disease progresses, thus investigating risk factors and predictive model for CI may contribute to a better understanding of the PD-RBD phenotype and help decelerate the MCI process in PD-RBD patients in clinical practice.

The central nervous system (CNS) is so sophisticated and fragile that is vulnerable to injury, pathogen, or neurodegeneration. In the process of aging and degradation, the activation of the innate immune system occurs. The inflammasome is a signal complex that regulates immune cells, chiefly microglial, leading to the release of pro-inflammatory factors. Consequently, pro-inflammatory factors aggravate the neuro-inflammatory response, neurological degeneration, and eventually upset the brain metabolic balance. It can be deduced that analyzing the inflammatory factors in CSF is more meaningful to investigate brain neuroinflammation. For instance, studies have shown that immune dysfunction may lead to aberrant α-Syn aggregation and transmission in the brain. The article by Lian et al. tackles this issue and suggests that neuroinflammatory factors in CSF, especially tumor necrosis factor (TNF)-α, may cause damage to dopamine (DA) neurons and induce the depletion of DA, which is related to the occurrence and development in PD patients who accompanied with depression. Also, adaptive immune system, including lymphocyte subpopulations and cytokines, is involved in the PD pathogenesis. The article by Huang et al. analyzes integrated substantia nigra (SN) and gene expression by CIBERSPOT and identifies the function of key immune cell types in PD. They propose the immune response modifying the genetic background as a factor influencing the synaptic pathway in PD pathology. Another popular inflammation topic normally discussed in pathogenesis of neurodegenerative disease is gut-brain axis. The gut-brain axis is a two-way regulating axis for the interaction between gastrointestinal function and the CNS, and the nerve-endocrine-immune network is an important connection method. Following this clue, Bu, Huang, et al. assess α-Syn immune-neuron reactivity and expression patterns. Contrary to our expectations, they propose that gastrointestinal pathology couldn't be facilitated for diagnosing α-synucleinopathies, including PD. The deposit of α-Syn is age-dependent rather than neurodegenerative-unique in the enteric nervous system (ENS) of patients.

Apart from aging, genetic dysfunction is widely considered to be a key risk factor of PD, and is repeatedly discussed when exploring the etiology and pathology of α-Syn. So far, at least 23 genes and independent risk signals related to PD have been discovered. Among them, it is indisputable that the α-synuclein gene (*SNCA*) is the first identified pathogenic gene that causes autosomal dominant PD, and its abnormal protein aggregation is regarded as the main pathological symbol of patients. Based on this understanding, Guo et al. evaluates the associations between the *SNCA* gene variants and PD through conventional whole-exome sequencing (WES) and sanger sequencing. Beyond expectations, the result shows that variants located in the coding regions of the *SNCA* gene may have less or no role in the development of PD in the Han Chinese population, which implies the inescapable role of ethnic differences in PD development. Therefore, in order to verify the function of *SNCA*, it is necessary to carry out cohort comparisons with large samples of multiple races in the future. Neuro regulation is the result of multi-gene collaboration. Both α-Syn and Monoamine oxidase A (MAOA) are long deemed as the pivotal pathogenesis of PD, Jia et al. further explores the close crosstalk between these two co-regulation molecules. At the same time, Bu, Xie et al. reveal that overexpression of long non-coding RNA (lncRNA)-T199678 mitigates the α-Syn-induced dopaminergic neuron injury, including oxidative stress, abnormal cell cycle, and apoptosis, which contributed to promote PD. This study praises a new molecular target for further genetic pathway investigation in PD.

Multiple system diseases often occur in the elderly. It is often found that patients with neurodegenerative diseases are complicated by osteoporosis, and fractures are more likely to be found in the prodromal phase of PD in clinical practice. As the main supporting and protecting organs of our body, bone is also an active immune and endocrine organ. It can secrete a variety of bone-derived factors, interact with the brain through various bone-derived cells, and participate in the pathophysiology of various diseases. First, the article by Yu et al. reviews the latest research studying the impact of bone-derived cells and bone-controlled immune system on the brain, summarizes the roles of the bone-brain axis in the progression of neurodegenerative diseases and evaluates how these factors are implicated in the progress of α-synucleinopathies diseases and their potential use in the diagnosis and treatment of these diseases. On the other hand, Lin et al. guaranteed the combined assessment of plasma and CSF of osteocalcin (OCN) or osteopontin (OPN) could be candidate biomarkers in differentiating PD patients from HCs. Besides, inflammation may be involved in the interaction between these two bone-derived factors and PD, which partly explains the various modulating effects of bone physiological processes on the CNS.

New methods and molecules are gradually being tried for scientific research. Recently, exosomal biomarkers have been involved in embedding the acknowledgment and diagnosis of neurodegenerative disorders. Exosomes are small extracellular vesicles coming from different cell types and can contain different molecular components such as proteins, lipids, and RNA. Previous reports have demonstrated increased exosomal α-Syn in patients with PD in comparison to HCs. In this context, the exosomal acetylcholinesterase (AChE) and α-Syn are quantified and the inner relationships with clinical symptom are simultaneously scouted by Shim et al. supporting the occurrence of cholinergic dysfunction on motor deficits in PD. The mechanisms of progressive loss of DA neurons in PD include a variety of cellular processes such as α-Syn aggregation, mitochondrial damage, oxidative stress, calcium homeostasis dysfunction, interruption of axonal transport, and neuroinflammatory damage. These abnormal phenomena can disturb the balance of protein homeostasis in PD. In a review by Ren et al., the cross-linking mechanisms of endoplasmic reticulum (ER) stress-induced unfolded protein response (UPR) and the dysregulated autophagy in DA neurodegeneration are systematically addressed.

Neuroimaging is also a traditional technique for detecting the connectivity of brain functions, which is often used in interpreting the static state and dynamic changes of patients in clinical research. Focusing on the brain imaging network in aging, it is wildly acknowledged that PD patients depict abnormal spontaneous neural activity in resting-state functional magnetic resonance imaging (r-fMRI). Wang Z. et al. target at the amplitude of low-frequency fluctuations (ALFF) and discover that PD patients exhibited different tendencies in several brain regions, emphasizing the diversity of widespread abnormal brain activities related to PD, and suggesting that the abnormalities of ALFF in specific frequency bands would be helpful in detecting the neural changes in PD. Expect for traditional neuroimaging, nowadays researchers use more controllable electrophysiological tools, such as TMS, to reveal the conduction blocks caused by structural abnormalities or neurodegenerative diseases. The article by Xu et al. applies a more sensitive and accurate method, triple stimulation technique (TST), to quantitatively assess the integrity and impairment of corticospinal pathway in PD patients.

Present research regards the electroencephalogram (EEG) as a promising method to study real-time brain dynamic changes in patients with PD. Compared with other functional imaging methods, EEG has high temporal resolution, in which specific frequency analysis enables accurately investigating connectivity fluctuations. Firstly, the review by Wang Q. et al. summarizes recent research on EEG characterization related to the cognitive and motor functions in PD patients and discussed its potential to be used as diagnostic biomarkers. Abnormalities in β and δ frequency bands are, respectively the main manifestation of dyskinesia and cognitive decline in PD. In conclude, the unique time-frequency characteristics of EEG could provide further insight into disrupted sensorimotor networks in PD with motor deficits, such as FOG, dyskinesia, and dystonia in a real-world environment. Several of the contributors to this topic recognized the need to promote cognition of older people. The exploration of specific brain regions for PD cognitive impairment is ongoing. The dorsolateral prefrontal cortex (DLPFC) transmits afferent projections to the caudate nucleus and putamen and participates in advanced cognitive functions, which is recognized as an important area related to CI. Furthermore, Cai et al. compare the posterior middle frontal gyrus-based functional connectivity differences between PD patients with MCI and normal cognitive PD patients. As discussed previously in this editorial, this topic accumulates growing evidence that cognitive dysfunction of PD patients is different from the cognitive function of normal aging people.

In summary, PD is a neurodegenerative disease caused by the combined action of genes, age, and environment. The underlying pathological mechanism involves multiple circuits such as gene regulation, neuroimmune, biochemical factors, and neurophysiology, which requires further exploration by establishing multi-factor model. On the other hand, biomarkers can help clinicians reduce the chance of misdiagnosis and help them monitor the improvement of disease pathology during anti-Parkinson's disease drug trials. However, the inconsistent research conclusions in the same direction remind us that many methodological issues related to the detection and quantification of α-syn need to be further verified for their feasibility. At the same time, large sample of long-term follow-up studies comparing the motor dysfunction of different neurodegenerative diseases are needed.

## Author Contributions

JL and PL decided on the basic framework of the editorial, and PL wrote the initial version of the manuscript. Also, JL and PX revised the work. All the authors approved of the final edition for publication.

## Conflict of Interest

The authors declare that the research was conducted in the absence of any commercial or financial relationships that could be construed as a potential conflict of interest.

## Publisher's Note

All claims expressed in this article are solely those of the authors and do not necessarily represent those of their affiliated organizations, or those of the publisher, the editors and the reviewers. Any product that may be evaluated in this article, or claim that may be made by its manufacturer, is not guaranteed or endorsed by the publisher.

